# French cross-cultural adaptation and validation of the Quality of Life-Alzheimer's Disease scale in Nursing Homes (QOL-AD NH)

**DOI:** 10.1186/s12955-021-01853-2

**Published:** 2021-09-15

**Authors:** Christophe Cousi, Valérie Igier, Bruno Quintard

**Affiliations:** 1 CLESCO ED 326, Centre for Studies and Research in Psychopathology and Health (CERPPS), University of Toulouse Jean-Jaurès, Toulouse, France; 2 INSERM BPH, UMR 1219, Team “Handicap, Activity, Cognition, Health”, University of Bordeaux, Bordeaux, France

**Keywords:** Quality of life, Dementia, Alzheimer’s disease, Nursing homes, QOL-AD

## Abstract

**Background:**

No specific scale to measure Quality of Life in Alzheimer's Disease in Nursing Homes (QoL-AD NH) exists in French. We aimed to translate and culturally adapt the QoL-AD NH participant scale into a French version and evaluate its psychometric properties with residents in French nursing homes (EHPAD).

**Methods:**

First, the QoL-AD NH was cross-culturally adapted into French according to guidelines. Secondly, a convenience group of residents with mild to moderate dementia answered the Folstein’s test and the QoL-AD NH. They also answered the Dementia Quality of Life and the Geriatric Depression Scale to test convergent and divergent validity. Known-group validity was tested with a comparison group of residents without dementia. Exploratory Structural Equation Modeling (ESEM) was used after Exploratory Factor Analysis (EFA) to identify factors and measure invariance across age and mental state groups. Reliability (internal consistency, McDonald’s omega and test–retest) were also measured.

**Results:**

Following successful adaptation of the QoL-AD NH, 174 residents (mean age 86.6) from 7 nursing homes with mild to moderate dementia participated in the validation study. We retained a 3-factor model of the scale after ESEM identifying: “Intra & interpersonal environment-related QoL”, “Self-functioning-related QoL” and “Perceived current health-related QoL” that were invariant across age and mental state groups. The QoL-AD NH had acceptable convergent (ρ range 0.24–0.53) and divergent validity (ρ range − 0.43 to − 0.57) and good known-group validity with 33 residents without dementia (*t*(205) = 2.70, *p* = .007). For reliability, the results revealed very good and adequate internal consistency (α = 0.86 for total scale and ≥ 0.71 for subscales). All total omega values exceeded the threshold 0.70. The hierarchical omega was 0.50, supporting the multidimensionality of the scale. Hierarchical omega subscale values exceeded the minimal level 0.50 except for the third factor, although reliable, would deserve more items. Test–retest was good with ICC (3,1) = 0.76.

**Conclusions:**

The QoL-AD NH French participant version has globally good reliability and validity for evaluating residents' quality of life. However, further studies must rework and confirm the factor structure, test sensitivity to change and responsiveness.

**Supplementary Information:**

The online version contains supplementary material available at 10.1186/s12955-021-01853-2.

## Background

In France, nursing homes for elderly (EHPAD—*Établissement d’Hébergement pour Personnes âgées Dépendantes*) are accommodating increasingly older residents with chronic illnesses and major cognitive impairments. More than 70% of EHPAD residents can have major cognitive impairments [1–3]. Knowing that the average length of stay of a resident in a nursing home is 3.6 years [4], evaluating and improving quality of life in nursing homes is a critical societal issue. In this way, the French National Agency for the Evaluation and Quality of Social and Medico-social Establishments and Services (ANESM) has included an approach focused on Quality of life in EHPAD in its work program [5]. In strand 1 of this program, the ANESM declares (p. 7):

An EHPAD is a place where the aim is the quality of life of each resident throughout their stay, whatever their difficulties: physical dependence, loss of decision-making autonomy, or difficulties in expressing themselves.

The concept of quality of life (QoL) has been conceptualised and defined differently, making it difficult to measure. In 1994, the World Health Organization defined QoL as:

An individual’s perceptions of their position in life in the context of the culture and value systems in which they live, and in relation to their goals, expectations, standards and concerns.

In the context of elderly cognitive impairment, there is emerging agreement on the meaning of QoL [6–8]. Lawton's model is the most widely used conceptualisation of QoL in Alzheimer's disease (and related disorders), including both objective and subjective factors based on four fundamental dimensions: “Psychological well-being” (e.g., positive and negative affects), “Behavioural skills” (e.g., cognitive and functional abilities), “Objective environment” (caregivers, helpers and living situation) and “Perceived QoL” [9]. According to this model, self-report would be preferable when feasible, and proxy-reports would occur when not feasible. However, a recent systematic study advocated the use of a proxy observation tool, the QUALIDEM, to assess the QoL of residents with cognitive impairments [10], although families and caregivers have been reported to undervalue the QoL of the person with cognitive impairment [11–15]. Nevertheless, despite the presence of cognitive impairment, many studies have shown that patients with Alzheimer’s disease were fully able to assess their QoL [11, 16–18], even up to an advanced disease stage [12, 19–21].

Over the past twenty-five years, researchers have developed many scales to assess HRQoL specific to dementia, yet there is not yet a gold standard to measure QoL in Azheimer’s disease [14, 22]. One scale, widely used in studies, is the QoL in Alzheimer’s Disease scale (QoL-AD 13) [23–25]. This scale is available in many European countries after cross-cultural adaptations [26–30], including France [31]. However, this scale may not be optimal for assessing QoL of residents who live in institutions as some items are specific to assess QoL at home. That is why Edelman et al. [13] slightly modified this tool to measure more precisely QoL in Alzheimer’s disease in elderly institutions/nursing homes with participant and proxy versions. While some researchers have used the QoL-AD NH scale in both versions (self and proxy-rated) [11–14, 32] or self-rated version only [33], others have only focused on proxy evaluation [15, 34–37] like Edelman et al. [13] who used the QoL-AD NH in a proxy evaluation with staff for the first time.

Several recent systematic reviews have emphasised weak or unexplored psychometric properties of dementia-specific QoL instruments for use in care settings, including the QoL-AD NH [10, 22, 24, 38]. Indeed, almost all studies have used the QoL-AD NH as an outcome measure but have never tested psychometric properties of the participant’s version. Only two studies have examined dimensionality of the QoL-AD NH, using only 11 items out of 15 on the scale [27, 39]. As a result, the QoL-AD NH scale has not yet shown real psychometric qualities, even in its original English version since there is no psychometric validation study. Finally, while there are scales that assess the HRQoL among community-dwelling older people in France [31, 40], there is, to our knowledge, no specific self-rating scale to assess residents’ QoL in Alzheimer’s disease in France, and more globally in the French-speaking world.

Our research aimed to provide a French cross-cultural adaptation and a first psychometric validation of the QoL-AD NH Participant version for French nursing home residents with mild to moderate Alzheimer’s disease or related disorders.

## Methods

### Study design

We led a descriptive cross-sectional study in two phases. In phase 1, the translation and cross-cultural adaptation of the QoL-AD NH (Participant version during an interview) from English into French was undertaken. In phase II, psychometric validation of the French adaptation was performed.

Ethical approval was obtained from the CERNI Ethics Committee of the University of Toulouse (number 2017-064).

### Phase 1: Translation and cross-cultural (transcultural) adaptation of the QoL-AD NH participant version

According to cross-cultural adaptation guidelines, a French adaptation was carried out, which involves adaptation, not just translation [41]. Several steps were necessary.

#### Translation (from English to French)

First of all, two French native-speaking translators independently translated the original English version of the QoL-AD NH; one of the translators (T1) was familiar with the study's concepts and the medical environment, the other (T2) was not.

#### Synthesis

A synthesis of the two translations was carried out, and a consensus was reached to develop a T-12 version.

#### Back translation (from French to English)

Working from the T-12 version of the scale, two English mother-tongue translators who were naive to the concepts and the medical environment carried out the back translation and produced B1 and B2 versions.

#### Expert committee review

After the translations, an expert committee met with the four translators, the principal investigator (CC) and the two co-authors (BQ, VI); the first one was specialised in QoL, the other in gerontology. This expert committee produced a pre-final version of the scale. The role was to consolidate all the translated versions considering four aspects: (1) semantic equivalence, (2) idiomatic equivalence, (3) experiential equivalence, and (4) conceptual equivalence.

#### Pretesting

The aim was to individually test the comprehension of the items and instructions of the adapted interview guide as well as the understanding and clarity of the response system. Ultimately, the aim was to assess the extent to which residents with mild to moderate cognitive impairment could complete the questionnaire according to the instructions and the proposed response system before use in the psychometric validation study.

Both Rebecca Logsdon (the original author and who participated in this French adaptation) and Perry Edelman (the lead author of the English nursing home adaptation) approved our cross-cultural adaptation into French.

### Phase 2: Psychometric validation of the QoL-AD NH French Participant version

#### Participants

Participants were residents of several nursing homes from the southwest of France.

They were recruited in two convenience samples according to different inclusion and exclusion criteria.

Inclusion criteria for the main sample were:

- To be at least 60 years old and a native French speaker
- To be a resident in the nursing home for more than three months
- To have a major neurocognitive disorder from mild to moderate stage
- To have a Mini-Mental State Examination (MMSE) score ≥ of 10

Exclusion criteria for the main sample were:

- Residents with major hearing or visual impairments despite the correction
- Residents hospitalised in the 30 days before the interview
- Residents with excessive cognitive-behavioural problems precluding answering the questions.

A comparison group of residents without cognitive impairment was also recruited. Criteria for non-cognitive impairment were confirmed by taking age and education into account in the MMSE score [42] and with the study group's first two inclusion/exclusion criteria.

The main sample size was based on the recommendations proposed by the Consensus-based Standards for the Selection of Health Measurement Instruments (COSMIN) to have at least five to ten times more participants per item of the questionnaire [43]. For this purpose and security purposes, the minimum estimated sample was 150. The sample size for the comparison group was set at a minimum of 30 participants and 25 participants for test–retest reliability (randomly extracted from the main group).

#### Questionnaires

A sociodemographic questionnaire indicated gender, age, education level, family situation, type of dementia, and dependency level, from GIR 6 (complete autonomy) to GIR 1 (totally dependent). This information was collected from the residents' medical records. Moreover, we noted any possible legal protection measure.

The protocol administered included the following questionnaires:

- The Mini-Mental State Examination (MMSE) [44]
  This scale assesses cognitive abilities, from no cognitive impairment to very severe cognitive impairment [45]. We used the GRECO version adapted and calibrated in French [42, 46]. In the absence of evidence to determine an MMSE cut-off score between mild and moderate cognitive impairment, two groups were created for comparison, taking as cut-off value the median of the MMSE scores [27]. Scores can range from 0 to 30 (higher scores indicating better cognitive status). MMSE scores were obtained either from the nursing home tests if run less than three months ago or as part of the study protocol. We did this to include residents in the right group according to their most current cognitive score.
- The QoL in Alzheimer’s Disease Nursing Home (QoL-AD NH) participant version
  The QoL-AD NH is an adaptation of Logsdon et al.’s [23] QoL-AD scale. The QoL-AD scale was adapted to QoL-AD NH [13] to assess the QoL in Alzheimer’s disease for residents in nursing homes. We used the adapted “Participant version” run directly with the resident answering questions during an interview. According to the original author of the QoL-AD 13 [23] and the QoL-AD NH [13], patients with an MMSE score ≥ 10 can complete the questionnaire. The scale includes 15 items rating from 1 to 4 points each: 1 (Poor); 2 (Fair); 3 (Good); 4 (Excellent) and total scores ranging from 15 to 60. Questionnaires with a maximum of two missing items were considered valid.
- Dementia Quality of Life (DQoL)
  The Dementia QoL (DQoL) scale was validated in English with a sample of 99 patients with dementia [16] and was adapted into French with elderly people with mild to moderate dementia [47]. This tool measures the construct of perceived QoL in Alzheimer’s disease of the elderly. It consists of 29 items on a 5-level Likert scale and covers five factors: (1) “Self-esteem” (2) “Positive affect/Humor” (3) “Negative affect” (4) “Feelings of belonging” (5) “Sense of Aesthetics”. A final optional item assessing the overall QoL was not used in this study. An average score was calculated for each DQoL dimension. This scale was recommended by a systematic review to assess QoL of residents with cognitive impairments [48]. So, we chose this scale to test convergent validity.
- The Geriatric Depression 15-item Scale (GDS-15)
  The GDS-15 assesses depressive complaints and the intensity of depression in the elderly [49, 50]. This scale validated in French [51] was well suited to older people without cognitive impairment and older people with mild to moderate cognitive impairment [52]. GDS-15 is rated yes/no. The minimum score is 0, and the maximum is 15. The higher the score, the more depressive symptoms and intensity are present. We chose this scale to test divergent validity.

#### Data collection

Twenty-one nursing homes (EHPAD) in France's southern Aquitaine region were initially contacted by phone to interview nursing home residents. The initial contact was often made with the psychologist of the EHPAD, who informed the nursing home director of the study and requested their participation. We clearly explained that the study only evaluated the perceived (and therefore subjective) resident’s QoL and not the care or facility's quality.

When the structures agreed, nursing home management initially informed residents and families that research would be conducted within the facility on the QoL of EHPAD residents. Taking into account potential participant vulnerability, we obtained the agreement of the curator when the resident was under legal protection. The research protocol was conducted on the study site exclusively by the same person (CC) who had good experience with residents and could give them the necessary time and support before, during and after the research protocol.

The recruitment of residents was first carried out with the help of the health care teams. They drew up an initial list of residents likely to be able and willing to respond to the questionnaire and according to the inclusion/exclusion criteria. The residents' medical and administrative records were consulted to confirm the caregivers' indications for Alzheimer disease (or related disorders) and collect the sociodemographic information. The data was then made anonymous.

Residents were informed of the study and individually invited to participate of their own free will in the research by asking for their agreement and when and at what time they wished to answer the questions. Before the interview, a simplified and large-print information and consent form was presented to residents explaining the aim of the research, the approximate duration, and data confidentiality and anonymity for aggregate results for scientific publication with a poster feedback of the simplified results that would be offered to them. They were also informed that they could stop the interview at any time and stop it permanently or resume it later if they so wished. Finally, we obtained their written consent signed in duplicate, one copy of which was given to the resident. The interview took place in their rooms.

The data collection was conducted from January to February 2020 before the Covid-19 pandemic and subsequent lockdowns.

#### Statistical analysis

##### Descriptive statistics

Descriptive statistics were presented as percentages, mean (± SD)/Range, age, type of dementia, MMSE score, gender, marital status, educational status, and GIR (level de dependency).

##### Acceptability

At first, the QoL-AD NH acceptability was assessed by the number of refusals and current dropouts and then by the percentage of missing data (rate of completed items) [53]. Floor and ceiling effect were analysed with a permissible limit of 15% [43]. Lastly, the administration time was calculated.

##### Normality test

Variables were assessed for univariate and multivariate normality. For skewness, if data were greater than + 1 or lower than -1 and for kurtosis data were greater than + 1, then data distribution essentially deviated from a normal distribution [54]. Multivariate normality for QoL-AD NH was evaluated using the Mardia test with a critical ratio below 8.00, indicating multivariate normality [55]. To identify univariate outliers, the variables were standardised. The cases with values exceeding 3.29 were considered outliers [56]. To evaluate multivariate outliers, the values of "Mahalanobis distance" were generated. The presence of possible outliers moving away from "centroids" and whose Mahalanobis *D*^2^ values were lower than 0.001 were considered multivariate outliers [56].

##### Factor analysis

We followed a two-step approach based on exploratory structural equation modeling (ESEM) [57]. ESEM can be seen as a compromise between the flexibility of EFA and the rigour of SEM [58]. While its application lies mainly in confirmatory research, ESEM has been used when factor structures were not yet well established [59, 60]. First, exploratory factor analysis (EFA) was conducted to determine factor loadings. The resulting loading matrix was then used to formulate a structural equation model (SEM), allowing for a more detailed model fit assessment.

Parallel Analysis (PA) was used to determine the optimal factors to extract [61]. We simulated 1000 random datasets. In addition to the model proposed by the parallel analysis, we fitted different EFA models that assumed one, two, or three factors. Fit indices of these models were compared in the ESEM step.

Exploratory factor analyses were conducted using maximum likelihood estimation and goemin rotation and an epsilon value of 0.50 [57, 62]. Geomin is an oblique rotation method, so the extracted factors could be correlated.

Once all EFA models were fit, ESEM was performed using their implied loading matrices. As mentioned above, ESEM was used to provide further guidance for selecting an adequate factor structure [63]. As cut-off criteria for good model fit, we mainly used the values provided by Hu and Bentler [64]: a model will be accepted if the Tucker-Lewis Index (TLI) surpasses 0.94 and the standardised root mean square residual (SRMR) is below 0.09.

Three criteria were taken into account to choose a model from our exploratory analysis: the interpretability of the factors, the model's simplicity, and the fit indices.

##### Measurement invariance

Measurement invariance was tested between three age groups: persons of 60 to 84 years of age (*n* = 50), 84 to 90 years (*n* = 60), and 90 to 100 years (*n* = 64). Measurement invariance was also tested between two groups by cognitive level (MMSE) with similar sizes: residents with MMSE scores below the median value (19), *n* = 90, and residents with MMSE scores above than the median value, *n* = 84.

We estimated a series of models with increasing equality constraints. The decline in model fit could easily be examined. If the difference in model fit after increasing constraints decreased too much, it could be concluded that measurement invariance did not hold. Following the cut-off scores proposed by Cheung and Rensvold [65], measurement invariance does not hold if the CFI drops by more than 0.01.

##### Convergent and divergent validity

Convergent validity was assessed using Spearman’s Rhô correlation comparing the QoL-AD NH with the DQoL. Divergent validity was also assessed using Spearman’s Rhô correlation, comparing the QoL-AD NH with the GDS-15. We retained the following values: weak (ρ < 0.25), moderate (0.25 < ρ < 0.50), good (0.50 < ρ < 0.75), and excellent (ρ > 0.75) [66]. We formulated four hypotheses inferred from the literature: 1) The total score of the QoL-AD NH would correlate positively and moderately with the scores of the four positive dimensions of the DQoL scale. 2) (If multifactor solution) The scores of the retained factors of the QoL-AD NH would correlate positively and moderately with the scores of the four positive dimensions of the DQoL scale. 3) The total score of the QoL-AD NH would correlate negatively and well with the score of the GDS-15. 4) (If multifactor solution) The scores of the retained factors of the QoL-AD NH would correlate negatively and well/moderately with the score of the GDS-15. Convergent and divergent validity were confirmed if at least 75% of the results corresponded with the hypotheses [43].

##### Known-group validity

The objective was to discriminate across groups known to differ theoretically [67]: residents with cognitive impairment and those without. Known-group validity was evaluated using an Independent Samples t-Test to test QoL scores between two groups. Secondly, a one-way ANOVA followed by a post hoc Bonferroni test to assess QoL scores between three groups (residents without cognitive impairment, mild cognitive impairment and moderate cognitive impairment). Based on the literature, we hypothesised that the group with dementia would have significantly lower scores of QoL than the residents without cognitive impairment. We also assumed that there would be a significant difference in QoL scores between the three groups (without, mild and moderate dementia). Known-group validity was confirmed if at least 75% of the results corresponded with the hypotheses [43].

##### Reliability analysis

Internal consistency. Internal consistency was assessed with Cronbach’s alpha. We used Kline's Cut-offs with coefficients around 0.90 considered “excellent,” values around 0.80 as “very good,” and values from 0.70 as “adequate” [68].

McDonald’s total omega, hierarchical omega, and hierarchical subscales. In multidimensionality, Cronbach’s alpha values often underestimate the reliability of scales [69]. Coefficient omega [70] allows to model sources of variance at general and multidimensional levels and is the more suitable estimate of reliability in the case of hypothesised multidimensionality [69, 71–73]. The bifactor model with the general QoL factor and multidimensional factor (factors determined by EFA/ESEM) was used to estimate omega values [69, 73, 74]. Three different omega values (total, hierarchical, and hierarchical subscales) were estimated to examine the factor solution received at EFA/ESEM stage. Total omega was estimated for the overall scale and three subscales. Total omega values above 0.70 indicate an acceptable level of composite reliability [75]. Hierarchical omega shows the proportion of variance attributable to a general factor [69]. There is no commonly accepted cut-off for hierarchical omega. A value above 0.50 is recommended as a low-level hierarchical omega, indicating that the scale is a valid instrument to measure a general factor [71]. The hierarchical omega subscale indicates the proportion of subscale scores variance explained by the multidimensional (subscale) factor after controlling for the effect of the general factor [76]. The value of the hierarchical omega subscale above 0.50 is considered enough for the reliability of a subscale [71].

Test–retest analysis. Test–retest reliability was assessed in a two-week interval in a group of twenty-five residents with mild to moderate cognitive impairment. In order to compare results with previous studies, we used both Pearson’s correlation and the Intraclass Correlation Coefficient of type ICC (3,1) with a 2-way mixed-effects model, single measurement, unique rater and absolute agreement. It was based on the following values: less than 0.50 (poor), between 0.50 and 0.75 (moderate), between 0.75 and 0.90 (good), and above 0.90 (excellent) [77].

All statistical analyses were done using R version 4.1.0 (2021–05-18) – "Camp Pontanezen", Brest, France [78] and the integrated development environment RStudio 1.4.1103 [79].

## Results

### Phase 1: Translation and cross-cultural adaptation of the QoL-AD NH French participant version

During the cross-cultural adaptation phase with the expert committee, some items had to be adapted, including item 09 “Personality overall” whose translation into “Self-image” was maintained (similar to the validated 13-item French version) [31]. Also, item 12 “Life overall” was adapted to “Current life in general” to clearly distinguish the desired meaning of current quality of life from life satisfaction. The supplementary document containing the instructions and scale questions for each item was also adapted. A pre-final version was developed and then pre-tested with twelve residents suffering from mild to moderate cognitive impairment who were not part of the sample for psychometric validation. This pre-final version proved to be well adapted and required only a few explanations for the item 09 “Self-image” and the item 15 “Ability to make choices in one’s life” with a slight rewording of the phrasing of the questions in the interview guide.

### Phase 2: Psychometric properties of the French QoL-AD NH French Participant version

#### Descriptive statistics

Of the twenty-one nursing homes contacted, seven agreed to participate, i.e. 1/3 of them. Four were private structures, and three were associations. The size and capacity of these structures varied from 35 to 90 residents. A total of one hundred and seventy-four residents with mild to moderate cognitive impairment participated in the research (Table 1) and a comparison group of thirty-three residents without cognitive impairment joined the study only to test known-group validity. Both groups had equivalent socio-demographic characteristics but the comparison group did not suffer from any cognitive impairment, with an average MMSE score of 26 (± 2.6) on a scale of 24 to 30.

**Table 1:** Description of nursing home residents with Alzheimer’s disease or related disorders (*n* = 174)

	*N*	%	Mean (± SD)/Range
Age in years	174	100.0	86.6 (± 7.6)/60–100
60–69	9	5.2	
70–79	16	9.2	
80–89	75	43.1	
90–100	74	42.5	
Type of probable dementia	174	100.0	
Alzheimer’s disease	138	79.3	
Mixed dementia	24	13.8	
Vascular dementia	12	6.9	
MMSE score			
Total score	174	100.0	18.8 (± 5.1)/10–26
< 19	78	81.6	13.9 (± 6.6)/10–18
≥ 19	96	18.4	22.7 (± 2.3)/19–26
Gender			
Female	142	81.6	
Male	32	18.4	
Marital status			
Single	54	31.1	
Married	11	6.3	
Divorced	4	2.3	
Widowed	105	60.3	
Educational status			
No qualification	46	26.4	
Primary diploma	80	46.0	
Secondary	29	16.7	
Higher education	19	10.9	
GIR*			
GIR 1	1	0.6	
GIR 2	39	22.4	
GIR 3	41	23.6	
GIR 4	72	41.3	
GIR 5	16	9.2	
GIR 6	5	2.9	

^*^GIR level of dependency from GIR 1 (the most dependant) to GIR 6 (the least dependant)

#### Acceptability

All invited participants accepted to participate and completed the protocol. The completion rate was 99.6% for the QoL-AD NH. No floor (0%) or ceiling effect (0%) was identified for the QoL-AD NH. With the presentation of the scale and the instructions, the average time taken to complete this questionnaire was 11 min.

#### Normality test

The Skewness of two items (item 4 & item 6) were just above -1. As a result, these variables were not normally distributed because there were negatively skewed. A correction log10(max(x + 1) − x) was applied to try to correct negatively skewed data with success. If the QoL-AD NH continuous variables were normally distributed, continuous variables from the GDS-15 and the dimension “Feeling of belonging” of the DQoL were not. The critical ratio of the Mardia test was 7.84, so there was multivariate normality [55]. There was no univariate or multivariate outlier [56].

#### Factor structure

Item 12 “Current life in general” was not included in the factor analysis because it was the general item of the scale and was identical to the Dementia Quality of Life Scale item that was not part of the factor structure but as an item evaluating and generally summarising the perceived QoL [16, 39]. Also, as it is a QoL in Alzheimer’s disease scale, only the 174 residents with Alzheimer’s disease or related disorders from mild to moderated cognitive impairment were included in factor analysis.

##### Parallel Analysis (PA)

A parallel analysis was conducted. Finally, four factors were suggested for exploratory factor analysis.

##### Exploratory Factor Analysis (EFA)

The Kaiser–Meyer–Olkin measure of sampling adequacy (KMO) was 0.84 and Bartlett’s test for sphericity was significant (χ^2^(91) = 699.34, *p* < 0.001), so we proceeded with the factor analysis. Four EFA models (models 1 to 4) with one to four factors were examined. Their goodness-of-fit statistics are displayed in Table 2.

**Table 2:** Comparison of the four models by their fit indices

	*χ*^2^	*df*	*p*	CFI	RMSEA	TLI	SRMR
One factor	193.52	90	0.000	0.821	0.085	0.819	0.082
Two factors	143.13	88	0.000	0.905	0.062	0.902	0.068
Three factors	88.50	85	0.390	0.995	0.015	0.994	0.051
Four factors	56.60	81	0.982	1.000	0.000	1.047	0.042

Χ^2^, chi-square; df, degrees of freedom; CFI, comparative fit index; RMSEA, root mean square error of approximation; TLI, Tucker–Lewis index; SRMR, standardised root mean square residual

##### Exploratory Structural Equation Modeling (ESEM)

The results showed that the one-factor and two-factor models did not fit. Only the three-factor and four-factor models had good fit indices (Table 2).

The four-factor model adjusted too much to the data indicating probable overfitting. Moreover, two of the four factors were based on only two items, and one of the other factors was difficult to interpret (Additional file 4). On the contrary, the three-factor model was easy interpretable, simple and a good fit to data. As a result, this three-dimensional model was chosen. Standardised factor loadings of this model are summarised in Table 3.

**Table 3:** Standardised factor loadings, eigenvalues, factor intercorrelations, and variance explained for 3-factor model

Item	Factor 1	Factor 2	Factor 3
Physical health	− 0.02	0.02	**0.89**
Vitality	0.09	0.10	**0.53**
Moral/mood	**0.50**	− 0.03	0.27
Living environment	**0.63**	0.07	− 0.09
Memory	0.12	**0.35**	0.01
Relationship with family	**0.44**	− 0.07	0.00
Relationship with staff	**0.63**	− 0.17	0.08
Relationship with friends	**0.46**	0.18	− 0.07
Self-image	**0.38**	0.16	0.16
Keep busy	0.02	**0.69**	0.08
Do things for pleasure	− 0.04	**0.68**	0.12
Self-care	**0.36**	0.13	0.15
Live with others	**0.43**	0.21	− 0.01
Make choices	0.19	**0.58**	− 0.07
Eigenvalues	4.79	1.36	1.20
Factor intercorrelations			
Factor 1	–		
Factor 2	0.47	–	
Factor 3	0.38	0.41	–
Variance explained	16%	13%	12%

Bold numbers represent the most appropriate items corresponding to each factor, as indicated by their maximum factor loading

The three factors were interpreted as follows: Factor 1: “Intrapersonal & interpersonal environment-related QoL”; Factor 2: “Self-functioning-related QoL”; Factor 3: “Perceived current health-related QoL” (Table 3).

#### Measurement invariance (MI) of the selected model

Measurement invariance analysis by age group showed no significant drop in model fit. We observed a comparative fit index (CFI) of 0.889 for the configural invariance model which remained constant in the metric invariance model. Finally, a drop of 0.003 was observed in the scalar invariance model [65]. It was concluded that the three-factor model was measurement invariant by age group (Table 4).

**Table 4:** Measurement invariance across age groups

	cfi.robust	rmsea.robust
Configural	0.889	0.073
Metric invariance	0.889	0.073
Scalar invariance	0.885	0.071

Regarding measurement invariance by mental state, we did not observe decreasing CFA and increasing RMSEA when comparing models. Also, fit CFA and RMSEA values indicated an acceptable fit for configural and constrained models [65]. Thus, the 3-factor model demonstrated measurement invariance across groups with lower and higher MMSE scores (Table 5).

**Table 5:** Measurement invariance across the mental status groups

	cfi.robust	rmsea.robust
Configural	0.922	0.059
Metric invariance	0.922	0.059
Scalar invariance	0.928	0.055

#### Convergent validity

The results showed that the QoL-AD NH Total score correlated positively and well/moderately with the four positive dimensions of the DQoL, “Sense of aesthetics” (ρ = 0.44, *p* < 0.01), “Positive affect” (ρ = 0.51, *p* < 0.01), “Feeling of belonging” (ρ = 0.46, *p* < 0.01), “Self-esteem” (ρ = 0.53, *p* < 0.01) thus confirming the hypothesis. Moreover, the three factors of the QoL-AD NH correlated positively with the four positive dimensions of the DQoL: Factor 1 score “Intrapersonal & interpersonal environment-related QoL” correlated positively and moderately/well with the four positive dimensions of the DQoL, “Sense of aesthetics” (ρ = 0.42, *p* < 0.01), “Positive affect” (ρ = 0.52, *p* < 0.01), “Feeling of belonging” (ρ = 0.49, *p* < 0.01), “Self-esteem” (ρ = 0.43, *p* < 0.01) confirming the hypothesis; Factor 2 score “Self-functioning-related QoL” correlated positively and moderately with the four positive dimensions of the DQoL, “Sense of aesthetics” (ρ = 0.38, *p* < 0.01), “Positive affect” (ρ = 0.39, *p* < 0.01), “Feeling of belonging” (ρ = 0.30, *p* < 0.01), “Self-esteem” (ρ = 0.43, *p* < 0.01) confirming the hypothesis; Factor 3 score “Perceived current health-related QoL” correlated positively and moderately/weakly with the four positive dimensions of the DQoL, “Sense of aesthetics” (ρ = 0.25, *p* < 0.05), “Positive affect” (ρ = 0.22, *p* < 0.05), “Feeling of belonging” (ρ = 0.24, *p* < 0.05), “Self-esteem” (ρ = 0.42, *p* < 0.01) partially confirming the hypothesis (Additional file 1: Table S1). These results revealed acceptable convergent validity [43].

#### Divergent validity

The QoL-AD NH total score was highly and negatively correlated with the GDS-15 scale score (ρ = -0.57, *p* < 0.01), confirming the hypothesis. Moreover, the GDS-15 score correlated negatively and moderately with the three factors of the QoL-AD NH: with Factor 1 score “Intrapersonal & interpersonal environment-related QoL” (ρ = -0.48, *p* < 0.01), with Factor 2 score “Self-functioning-related QoL” (ρ = -0.45, *p* < 0.01) and with Factor 3 score “Perceived current health-related QoL” (ρ = -0.43, *p* < 0.01) partially confirming the hypothesis (Additional file 1: Table S1). These results revealed acceptable divergent validity [43].

#### Known-group validity

First of all, we compared two groups: residents without cognitive impairment (*n* = 33) and the whole group of residents with cognitive impairment (*n* = 174). As predicted, QoL of the residents with cognitive impairment was significantly lower (37.4 ± 6.2) than the QoL of residents without cognitive impairment (40.5 ± 5.2), *t*(205) = 2.70, *p* = 0.007, thus confirming the hypothesis. Secondly, we compared the three groups of residents' QoL: residents with mild dementia (*n* = 96), moderate dementia (*n* = 78) and without cognitive impairment (*n* = 33). The results revealed a significant difference between groups determined by the one-way ANOVA, *F*(2,204) = 4.871, *p* = 0.009. A Bonferroni post hoc test showed that the residents' QoL with moderate cognitive impairment was significantly lower (36.7 ± 6.2, *p* = 0.006) than the QoL of residents without cognitive impairment (40.5 ± 5.2). However, there was no significant difference between the QoL of residents with moderate cognitive impairment and the residents with mild cognitive impairment (*p* = 0.379) and between the residents with mild cognitive impairment and residents without cognitive impairment (*p* = 0.129). As a result, the hypothesis was partly confirmed. Globally, these results confirmed good known-group validity [43]. Finally, we resumed means and standard deviations for each group's continuous variables (Additional file 2) and each QoL-AD NH scale item (Additional file 3).

#### Reliability analysis

##### Internal consistency

Cronbach’s alpha for all 15 items of the QoL-AD NH was α = 0.86. So, the internal consistency of the total scale was very good [68]. Once the three-factor model was selected after factor analysis and ESEM, we measured internal consistency for the subscales (Table 6). The results showed that the Cronbach’s alpha was very good for Factor 1 (“Intrapersonal & interpersonal environment-related QoL”, α = 0.77), adequate for Factor 2 and Factor 3 (“Self-functioning-related QoL”, α = 0.72; “Perceived current health-related QoL”, α = 0.71) [68].

**Table 6:** Cronbach’s alpha and McDonald’s total omega, hierarchical omega and hierarchical subscales for the three-factor model

	α	ω_s_(ω)	ω_hs_(ω_h_)
F1 “Intraperso. & interperso. environment-related QoL”	0.77	0.89	0.60
F2 “Self-functioning-related QoL”	0.72	0.84	0.58
F3 “Perceived current health-related QoL”	0.71	0.74	0.34
QoL total scale	0.86	(0.91)	(0.50)

α = alpha, ω_s_(ω) − ω_s_ = total omega for subscales and total ω for total scale, ω_hs_ = omega hierarchical for subscales, ω_h_ = omega hierarchical for general factor (general factor is a latent factor of QoL)

##### McDonald’s total omega, hierarchical omega, and hierarchical subscales (Table 6)

In the 3-factor QoL-AD NH scale, total omega values were high for both the total scale and subscale scores: 0.91 (Total scale), 0.89 (“Intrapersonal & interpersonal environment-related QoL”), 0.84 (“Self-functioning-related QoL”), and 0.74 (“Perceived current health-related QoL”). All total omega values exceeded the threshold 0.70 [75], supporting composite reliability of the scale in general and each of three subscales. The hierarchical omega was 0.50. When compared to the total omega value of 0.91, it is clear that the explained variance in the total QoL scores is attributable in approximately equal proportions to the general QoL factor (around 55%) and multidimensionality factor (around 45%). It indicates that raw QoL scores were essentially affected by the subscale factor supporting the multidimensionality of the QoL scale. Omega hierarchical subscale values exceeded the minimal level 0.50 [71] for “Intrapersonal & interpersonal environment-related QoL” (0.60) and “Self-functioning-related QoL” (0.58) subscales, indicating that the majority of subscale score variance was attributable to the multidimensional factor even after the removing effect of the general factor. The multidimensional factor contributed 67% and 69% in the total explained variance of subscale scores, indicating that the majority of reliable variance of these subscale scores was independent of the general factor. Hierarchical omega for the third factor “Perceived current health-related QoL” was 0.34, that was lower than the level 0.50. However, comparing this score with the total omega score of this subscale, 0.74, it indicates that the subscale factor contributed significantly (46%) to the total explained variance of subscale scores after controlling for the general factor.

##### Test–retest analysis

Finally, test–retest reliability was calculated by the Pearson’s correlation coefficient and the ICC (3,1). The results revealed good test–retest reliability at 15-day intervals, with *r*(23) = 0.89, *p* = 0.01, and ICC (3,1) = 0.76, *p* = 0.001 [77].

## Discussion

We aimed to provide a cross-cultural adaptation in French and the first psychometric validation of the QoL in Alzheimer’s Disease Nursing Home Scale—Participant version. To our knowledge, there was no specific scale to assess QoL in Alzheimer's disease for French-speaking residents in nursing homes. Moreover, the QoL-AD NH had not been thoroughly analysed, even in its original English language. Our global results show that after the cross-cultural process and study validation, the French adaptation of the QoL-AD NH Participant version has globally good psychometric properties with a three-dimensional factor structure (invariant across age and mental status groups), acceptable convergent and divergent validity, good known-group validity, adequate internal consistency/omega values and good test–retest reliability.

### Interpretation of the results and comparison with the literature

Since the psychometric qualities of QoL-AD NH have not been widely explored, we did not have sufficient theoretical elements to perform a Confirmatory Factor Analysis (CFA). To analyse the factor structure, we used both EFA and ESEM. The use of the ESEM combined the advantages of EFA and CFA and allowed us to select the most appropriate model while remaining in an exploratory design.

We finally retained the three-factor model, which was, at the same time, interpretable, simple and a good fit to data: Factor 1 “Intrapersonal & interpersonal environment-related QoL” (8 items); Factor 2 “Self-functioning-related QoL” (4 items); and Factor 3 “Perceived current health-related QoL” (2 items). These three factors seem to be representative of and pertinent to the resident's QoL in Alzheimer’s disease in nursing homes. Indeed, for Factor 1, relationships are essential for nursing home residents, especially with their families [80–82]. The more objective living environment also plays a vital role in residents' QoL with cognitive disorders, mainly when adapted to their disability and facilitates their taking temporospatial bearings [9, 81, 83]. About the “Self-functioning-related QoL” factor, it is very relevant as the resident can feel that he or she is still able to make choices, that he or she is given a choice, especially in daily activities [80, 82, 84]; this is what the person-centred care and the Montessori approach applied to the elderly advocate [85–87]. Finally, it is not surprising to find a perceived health factor in a health-related quality of life scale that usually contains physical and mental health measures with other elements not directly related to health [53, 88]. This factor is based on only two items. However, some health-related quality of life scales can have 2-item factors [89–91], and we discuss their reliability further. Perceived health has always been important, regardless of the person's age and level of cognitive impairment [80, 92]. Moreover, the phenomenon of anosognosia may allow some residents to be not fully aware of their state of health; this may be a protector as they often report having good physical health/vitality and good QoL [93–95].

Our results contrast to the initial Edelman’s study that showed a unique factor structure [13]. Indeed, in our study, the three-factor solution supports the multidimensionality of the concept of QoL in Alzheimer's disease as evoked by Lawton's model, including both objective and subjective factors [9]. Our findings also seem consistent with the Spanish validation led in day centres containing 11 of the 15 items of the QoL-AD NH, which revealed the same three-factor structure in the EFA step [27]. Nevertheless, after CFA, they decided to retain a two-factor solution even if the three-factor model fitted well [96]. Moreover, as in the Spanish study, we did not find the “Psychological well-being” factor evoked in a few validation studies of the QoL-AD 13 [97, 98]. However, our results are consistent with an Australian study conducted in long-term care facilities, revealing an identical three-factor structure [39]. This study aimed to extract a new classification system for economic evaluation from the QoL-AD NH factor structure. Based on 11 of the 15 items in the QoL-AD NH, an EFA and then a CFA confirmed a three-factor model.

Our factor analysis contained 14 of the 15 items due to excluding the global QoL item (item 12), as other studies have done [16, 39]. However, a few items loaded a bit low but were acceptable for an EFA and ESEM as they loaded at least 0.35, in agreement with previous studies and a part of the literature admitting a threshold of 0.30 or 0.32 [39, 56, 99, 100]. Moreover, there was no cross-loading because the difference between the highest loading and the second-highest loading for all items was greater than 0.20 [101]. For example, item 05 “Memory”, was identified in the literature as a low loading item [23, 27, 39, 97]. However, this item is essential to the scale because it is specific to Alzheimer’s disease (or related disorders) and probably more critical for a proxy evaluation of anosognosia. In that sense, we wanted to keep all the items on the one hand because the factorial results were only valid for our exploratory sample. Conversely, further studies are to be carried out to validate the hetero-evaluation version and compare the two versions. Indeed, in its original version, the author advocates calculating a composite score that would consist of 2/3 of the score on the resident's evaluation and 1/3 on the hetero-evaluation. In addition, as discussed below in the clinical recommendations, the individual consideration of items to assess change in the QoL would be of clinical significance and should be taken into account.

When we examined convergent validity, we found significant positive and moderate correlations between the QoL-AD NH and the DQoL. This may suggest that the QoL-AD NH captures further information, which is not included by the DQoL that is not specific to older people who live in a nursing home. However, the literature consistently showed significant but moderate positive correlations (i.e. around 0.30-0.63) between the QoL-AD 13 (from which QoL-AD NH derives) total score and the dimensions of the DQoL [29, 32, 102]. Also, when we examined the correlations between the three extracted factors from the QoL-AD NH and the four positive factors of the DQoL (Additional file 1), we found that the weakest correlations between the two scales lay between the “Perceived current health-related QoL” factor of the QoL-AD NH and the different factors of the DQoL. The perceived health factor that is based on 2 items may affect the correlation. However, this may be because the DQoL scale is considered as a needs assessment tool rather than an HRQoL tool [48]. The question arises as to whether the QoL-AD NH itself can be regarded as a purely HRQoL scale [39] when it originally seemed to inherit Lawton's model. While the QoL-AD was also classified as a Patient-Reported Outcome Measures (PROMs), the vast majority of the tools used in studies were proxy scales [103]. Concerning divergent validity, the factor “Perceived current health-related QoL” is the factor that correlates least with depression, with, even so, a moderately strong correlation. Our findings are similar to previous studies; there is a negative and significant relationship between QoL measured by the QoL-AD and depression [23, 26, 30]. Only one German study reports no relationship between QoL and depression [29]. However, many studies have shown that depression was one of QoL's strongest predictors in Alzheimer’s disease [21, 25, 33, 36]. We concluded that the convergent and divergent validities were globally aligned with the other studies and acceptable.

The results of known-group validity showed that the group of residents with cognitive impairment had significantly lower QoL scores than the residents without cognitive impairment. These results agree well with existing studies [26, 104]. However, taking into account the three groups of residents (without, mild and moderate cognitive impairment), the results showed that there was a significative difference in the QoL only between the group of residents without cognitive impairment and the group with moderate cognitive impairment, and therefore not with the group with mild cognitive impairment. This result may be due to the MMSE cut-off utilised to differentiate between groups, as we discuss further.

The question of whether the QoL-AD NH scale can also be used to assess QoL for residents without cognitive disorders is relevant. One study revealed the factor structure and measurement invariance of the QoL-AD 13 with a non-cognitive impairment community-dwelling sample [97]. This study showed a three-factor solution, including “Physical well-being”, “Social well-being” and “Psychological well-being”. Our study did not extract the factor “Psychological well-being” and we had only 9 common items in the scale; it is difficult to compare the results since it is not quite the same scale nor the same sample. However, we think the QoL-AD-NH could be used to assess QoL of residents without cognitive impairment, even if elements are necessarily missing in the QoL, such as spirituality that can be important for residents who do not have cognitive disorders [10, 48].

Lawton’s model, the most widely used theoretical model for conceptualising QoL in Alzheimer's disease, is not specific to Alzheimer’s QoL for people living in an institution but more generally to QoL in Alzheimer's disease. However, this model takes into account the objective environment, such as the living environment. In that sense, the institutional living environment in the EHPAD nursing home probably plays an essential role in the residents' QoL, beyond purely subjective factors. In our study, item 04 “Living environment” was the strongest explanatory component of factor 1 which itself was the factor explaining most of the variance of QoL-AD NH. While Lawton's model is still the most important conceptualisation of QoL in Alzheimer's disease, it would be appropriate to develop a more specific theoretical and conceptual framework of QoL in Alzheimer's disease in institutions, mainly in nursing homes.

Regarding the reliability of the QoL-AD NH, internal consistency was very good for the total scale and one subscale, adequate for the two others. Test–retest revealed a good ICC. These results are in line with other studies [13, 26, 27, 31]. Going further than Cronbach’s alpha by testing all McDonald’s omega values, the global results confirmed the reliability of the subscales. Testing McDonald’s hierarchical omega subscales, two subscales (“Intrapersonal & interpersonal environment-related QoL” and “Self-functioning-related QoL”) showed good reliability, contributing to subscale score variance irrespective of the general QoL factor (latent factor) influence. The third subscale “Perceived current health-related QoL”, had a less independent individual effect on subscale score variance than the general QoL factor (46% vs 64%). However, this effect is essential to consider that “Perceived current health-related QoL” is a reliable measure of the corresponding dimension of QoL. On the other hand, it indicates that this subscale requires future improvements, perhaps more items to increase reliability and, at the same time, validity.

### Strengths and limitations

In our study, the number of participants was more extensive than similar validation studies [26, 27, 29, 96]. Moreover, our psychometric validation went further than just EFA, using the ESEM method that combines the strengths of EFA and CFA while remaining an exploratory process. We also went beyond Cronbach's alpha by testing all MacDonald’s omega coefficients, including the omega hierarchical subscale. To our knowledge, this study is the first to examine the full validity and reliability of the QoL-AD NH, as the original version was never validated psychometrically. This study is also the first step towards the French adaptation and validation of a self-report scale that measures the QoL of nursing home residents with mild to moderate cognitive impairment.

Some study limitations should be acknowledged. First of all, we used EFA and ESEM on the same dataset; there may be a potential danger of overfitting [105]. However, we used ESEM to provide further guidance for selecting an adequate factor structure by staying in an exploratory design. Confirmatory studies will need to be conducted on another dataset. Secondly, it was difficult to determine a cut-off between mild and moderate cognitive impairment. The use of Folstein’s MMSE score as a criterion of categorisation between mild and cognitive impairment could have been combined with the Clinical Dementia Rate (CDR) scale [38] or the Global Deterioration Scale to refine the level of severity and cut-off in dementia. However, these scales were not validated in French. Thirdly, the measurement invariance results across age and mental state should be interpreted with caution as the sample size to test measurement invariance was small. This is the reason why we did not use measurement invariance as a criterion for selecting the factor model. Other studies could use measurement invariance as a criterion for selecting a factor model in ESEM if they have a sufficiently large number of participants. Lastly, Factor 3 “Perceived current health-related QoL” was composed of only two items and, although valid and reliable, could be reworked by adding a few items that could improve it.

### Future research and clinical recommendations

First of all, further research will have to carry out confirmatory factor analysis (CFA) of the QoL-AD NH and assess the proxy-report version's psychometric properties, adding validity and reliability to QoL-AD NH by comparing the self-report (during an interview) and proxy-report versions (by staff).

Researchers have long considered QoL as a secondary outcome in studies. However, QoL might, and should become, the principal outcome of interest in the coming years in nursing homes. There is a need to develop longitudinal studies and use repeated measures that are essential to assess sensitivity to change and responsiveness. However, we know the difficulty of QoL instruments in assessing significant changes in QoL measures. That is why from a clinical perspective and in agreement with Tractenberg and colleagues who compared the change in the measures of the QoL-AD 13, we think it is possible to leave the items of the QoL-AD NH as the individual QoL components to qualify the direction and quantification of this change [106]. For these researchers, accounting for change at the item level could provide more robust evidence of change in the QoL, including its improvement.

In this direction, it would be advisable to study and consider the “Response shift” phenomena. This change in response over time would consist of a change in internal standards (e.g. re-calibration), values (e.g. re-prioritisation) and reconceptualisation of what QoL can be like [107]. As a result, residents may perceive and interpret the questions put to them differently over time, depending on the course of their illness, in terms of meaning, priorities and impact on their personal life. While the response shift has been studied and taken into account with other HRQoL scales, it has never been studied in Alzheimer's disease. Consequently, other studies could try to ask the resident for each one of the items if it is “very important”, “moderately important” or “not important” for them. It would also be interesting to ask the resident's opinion for each item and monitor change if it is noticed from a clinical perspective.

Also, the QoL-AD NH could constitute a baseline for QoL of residents without cognitive impairment before the disease symptoms appear [97]. Moreover, after diagnosis, the QoL-AD NH could be used to assess the resident’s QoL and try to improve it, especially after a change in treatment or a non-drug intervention. Finally, staff could and should use the QoL-AD NH when drawing up the resident's life and care project in a dynamic co-construction approach involving the resident.

## Conclusions

The French adaptation of the QoL-AD NH has shown globally good psychometric properties in terms of both validity and reliability for the Participant version during an interview. There is now a specific French scale to assess residents' quality of life with mild to moderate cognitive impairment. However, a CFA and further longitudinal studies are needed to evaluate sensitivity to change and responsiveness of this scale. Enabling the residents with mild to moderate cognitive impairment to assess their own QoL is already the first step and could be the bridge to build towards a person-centred approach, a resident-centred approach in France.

## Supplementary Information

**Additional file 1: Table S1**. Spearman's correlation coefficient between the variables of the study (n = 174).

**Additional file 2: Table S2**. Mean, Standard deviation and Cronbach’s alpha of the continuous variables of each group study.

**Additional file 3: Table S3**. Groups mean and standard deviation for the 15 items of the QoL-AD NH.

**Additional file 4: Tables S4-S6.** Standardised factor loadings, eigenvalues, intercorrelations, variance explained and Cronbach’s alpha for one/two/four-factor ESEM model.

## Data Availability

QoL-AD NH (also named QoL-AD 15) is distributed by Mapi Research Trust on behalf of the copyright owner (Rebecca Logsdon): © 1996, Rebecca Logsdon, PhD; University of Washington. QoL-AD NH contact information, provision and permission to use: Mapi Research Trust, Lyon, France, https://eprovide.mapi-trust.org. The datasets used during the current study are available from the corresponding author on reasonable request.

## Abbreviations

ANESM: National agency for the evaluation and quality of social and medico-social establishments and services
CERNI: Non-interventional Research Ethics Committee
CFA: Confirmatory Factor Analysis
EFA: Exploratory Factor Analysis
EHPAD: Établissement d’Hébergement pour Personnes Agées Dépendantes
ESEM: Exploratory Structural Equation Modeling
HRQoL: Health-related Quality of Life
ICC: Intraclass Correlation Coefficients
PA: Parallel Analysis
PROMs: Patient-Reported Outcome Measures
QoL: Quality of Life
QoL-AD 13: Quality of Life in Alzheimer’s Disease 13 items
QoL-AD NH: Quality of Life in Alzheimer’s Disease Nursing Home version
SEM: Structural Equation Modeling
