# Supplementary material for: French cross-cultural adaptation and validation of the Quality of Life-Alzheimer's Disease scale in Nursing Homes (QOL-AD NH)
Source: Health Qual Life Outcomes. 2021 Sep 15;19:219. doi: 10.1186/s12955-021-01853-2 (PMC8443115; doi:10.1186/s12955-021-01853-2)
Supplement: Supplementary file 1 — Additional file 1: Table S1. Spearman's correlation coefficient between the variables of the study (n = 174). [file 12955_2021_1853_MOESM1_ESM.docx]

**Table S1** Spearman's correlation coefficient between the variables of the study (*n* = 174)

| Variables | 1 | 2 | 3 | 4 | 5 | 6 | 7 | 8 | 9 | 10 | 11 | 12 | 13 | 14 | 15 |
| --- | --- | --- | --- | --- | --- | --- | --- | --- | --- | --- | --- | --- | --- | --- | --- |
| 1 Age  2 Gender  3 Marital status  4 Level of education  5 GIR  6 MMSE Folstein  7 QoL AD NH Total 15 items  8 QoL-AD NH Factor 1  9 QoL-AD NH Factor 2  10 QoL-AD NH Factor 3  11 Depression (GDS-15)  12 DQoL Sense of aesthetics  13 DQoL Positive affect  14 DQoL Negative affect  15 DQoL Feeling of belonging  16 DQoL Self-esteem | .08  **.15^*^**  **.16***  -.03  .00  -.06  -.02  **-.15^*^**  .04  .01  -.06  -.10  .02  -.11  -.07 | .10  -.01  .03  -.03  .07  .05  .07  .07  -.06  -.04  -.03  -.14  -.12  -.09 | .12  **-.31****  **-.26****  -.11  -.13  -.08  .02  -.00  -.04  **-.32***  .15  -.13  -.06 | .04  **.19***  -.05  -.03  -.10  -.00  -.12  .07  -.05  -.13  -.01  -.02 | **.46^**^**  **.20****  .**24****  .10  .03  -.09  **.27***  **.29****  **-.29****  **.26***  .11 | .12  **.20****  .04  .05  -.01  **.23***  **.26***  **-.28****  .18  **.27*** | **.92****  **.81****  **.63****  **-.57****  **.44****  **.51****  **-.26***  **.46****  **.53**** | **.59****  **.45****  **-.48****  **.42****  **.52****  **-.24***  **.49****  **.43**** | **.44****  **-.45****  **.38****  **.39****  **-.24***  **.30****  **.43**** | **-.43****  **.25***  **.22***  -.09  **.24***  **.42**** | **-.23***  **-.39****  **.36****  **-.35****  **-.28**** | **.34****  -.04  **.41****  **.37**** | **-.41****  **.55****  **.46**** | -.17  **-.30**** | **.41**** |

^*^ *p* < 0.05. ^**^ *p* < 0.01; GIR: level of autonomy; QoL-AD NH: quality of life in Alzheimer’s disease nursing home version; GDS-15: geriatric depression scale 15 items; DQoL: dementia quality of life scale.
