# Supplementary material for: French cross-cultural adaptation and validation of the Quality of Life-Alzheimer's Disease scale in Nursing Homes (QOL-AD NH)
Source: Health Qual Life Outcomes. 2021 Sep 15;19:219. doi: 10.1186/s12955-021-01853-2 (PMC8443115; doi:10.1186/s12955-021-01853-2)
Supplement: Supplementary file 2 — Additional file 2: Table S2. Mean, Standard deviation and Cronbach’s alpha of the continuous variables of each group study. [file 12955_2021_1853_MOESM2_ESM.docx]

**Table S2** Mean, Standard deviation and Cronbach’s alpha of the continuous variables of each group study

| Item | Mild CI  *n* = 96 | | Moderate CI  *n* = 78 | | Mild + Mod. CI  *n* = 174 | | No CI  *n* = 33 | | Cronbach’s alpha  *n* = 174 | |
| --- | --- | --- | --- | --- | --- | --- | --- | --- | --- | --- |
| QoL-AD NH Total 15 items  Factor 1: Intra & Inter Envir.  Factor 2: Self-functioning  Factor 3: Perceived Health  Depression (GDS-15)  Dementia QoL (DQoL)  Sense of Aesthetics  Positive affect  Negative affect  Feelings of belonging  Self-esteem | 37.92 + 6.01  2.68 + .43  2.35 + .50  2.31 + .59  4.77 + 3.77  3.14 + .76  3.29 + .72  2.62 + .78  3.02 + .84  3.24 + .87 |  | 36.67 + 6.21  2.53 + .43  2.32 + .54  2.39 + .57  5.22 + 3.74  2.80 + .65  2.83 + .70  2.99 + .68  2.86 + .58  2.78 + .75 |  | 37.44 + 6.17  2.62 + .44  2.34 + .52  2.35 + .59  4.29 + 3.77  3.02 + .73  3.10 + .74  2.79 + .76  2.96 + .75  3.04 + .85 |  | 40.54 + 5.21  2.82 + .32  2.65 + .45  2.40 + .57  3.90 + 3.24  3.40 + .71  3.38 + .66  2.64 + .68  3.37 + .60  3.28 + .52 |  | .86  .77  .72  .71  .74  .71  .79  .87  .55  .79 |  |

CI: cognitive impairment; Mod: moderate; QoL-AD NH: quality of life in Alzheimer’s disease nursing home version; GDS-15: geriatric depression scale 15 items; DQoL: dementia quality of life scale.

Means for the DQoL factors are average scores (scores/number of items).
