# Supplementary material for: French cross-cultural adaptation and validation of the Quality of Life-Alzheimer's Disease scale in Nursing Homes (QOL-AD NH)
Source: Health Qual Life Outcomes. 2021 Sep 15;19:219. doi: 10.1186/s12955-021-01853-2 (PMC8443115; doi:10.1186/s12955-021-01853-2)
Supplement: Supplementary file 3 — Additional file 3: Table S3. Groups mean and standard deviation for the 15 items of the QoL-AD NH. [file 12955_2021_1853_MOESM3_ESM.docx]

**Table S3** Groups mean and standard deviation for the 15 items of the QoL-AD NH

| Items | Mild CI  *n* = 96 | Mod CI  *n* = 78 | Mild + Mod CI  *n* = 174 | Without CI  *n* = 33 |
| --- | --- | --- | --- | --- |
| 1 Physical health  2 Vitality  3 Moral, Mood  4 Living environment  5 Memory  6 Relationship with family  7 Relationship with staff  8 Relationship with friends  9 Self-image  10 Keep busy  11 Do things for pleasure  12 Current life in general  13 Self-care  14 Live with others  15 Make choices | 2.33 + .66  2.29 + .68  2.43 + .75  2.83 + .72  2.38 + .69  2.96 + .85  3.03 + .53  2.71 + .78  2.38 + .64  2.34 + .75  2.42 + .69  2.43 + .74  2.53 + .58  2.57 + .71  2.28 + .74 | 2.42 + .71  2.37 + .58  2.33 + .77  2.65 + .64  2.28 + .66  2.81 + .79  2.88 + .62  2.53 + .75  2.23 + .68  2.38 + .65  2.32 + .76  2.31 + .73  2.49 + .66  2.36 + .62  2.29 + .72 | 2.38 + .68  2.33 + .65  2.40 + .76  2.76 + .69  2.33 + .67  2.89 + .81  2.97 + .58  2.63 + .77  2.32 + .66  2.36 + .70  2.39 + .73  2.39 + .73  2.51 + .62  2.48 + .69  2.30 + .74 | 2.27 + .63  2.52 + .62  2.58 + .66  3.06 + .56  2.67 + .64  3.27 + .63  3.15 + .62  2.91 + .58  2.30 + .53  2.79 + .70  2.76 + .66  2.58 + .61  2.82 + .53  2.48 + .75  2.39 + .56 |

Mild CI: mild cognitive impairment; Mod CI: moderate cognitive impairment; Without CI: without cognitive impairment; QoL-AD NH: quality of life in Alzheimer’s disease nursing home version.
