# Supplementary material for: French cross-cultural adaptation and validation of the Quality of Life-Alzheimer's Disease scale in Nursing Homes (QOL-AD NH)
Source: Health Qual Life Outcomes. 2021 Sep 15;19:219. doi: 10.1186/s12955-021-01853-2 (PMC8443115; doi:10.1186/s12955-021-01853-2)
Supplement: Supplementary file 4 — Additional file 4: Tables S4-S6. Standardised factor loadings, eigenvalues, intercorrelations, variance explained and Cronbach’s alpha for one/two/four-factor ESEM model. [file 12955_2021_1853_MOESM4_ESM.docx]

**Table S4** Standardised factor loadings, eigenvalues, factor intercorrelations, variance explained and Cronbach’s alpha for 4-factor ESEM model

|  | Factor 1 | Factor 2 | Factor 3 | Factor 4 |
| --- | --- | --- | --- | --- |
| Physical health | 0.00 | **0.86** | -0.01 | 0.04 |
| Vitality | -0.02 | **0.54** | 0.21 | 0.03 |
| Moral / mood | **0.46** | 0.35 | 0.00 | 0.01 |
| Living environment | **0.68** | -0.04 | -0.04 | 0.12 |
| Memory | -0.06 | 0.02 | **0.65** | -0.02 |
| Relationship with family | **0.25** | 0.01 | 0.23 | -0.15 |
| Relationship with staff | **0.63** | 0.15 | 0.05 | -0.17 |
| Relationship with friends | **0.42** | -0.03 | 0.04 | 0.18 |
| Self-image | 0.12 | 0.14 | **0.51** | 0.07 |
| Keep busy | 0.07 | 0.07 | 0.00 | **0.81** |
| Do things for pleasure | -0.03 | 0.11 | 0.17 | **0.55** |
| Self-care | 0.15 | 0.24 | **0.42** | -0.03 |
| Live with others | 0.29 | 0.01 | **0.30** | 0.09 |
| Make choices | 0.01 | -0.12 | **0.48** | 0.35 |
| Eigenvalues | 4.79 | 1.36 | 1.20 | 1.03 |
| Factor intercorrelations |  |  |  |  |
| Factor 1 | - |  |  |  |
| Factor 2 | 0.28 | - |  |  |
| Factor 3 | 0.50 | 0.44 | - |  |
| Factor 4 | 0.31 | 0.35 | 0.41 | - |
| Variance explained | 12% | 11% | 12% | 10% |
| Cronbach’s alpha | 0.69 | 0.71 | 0.73 | 0.73 |

**Table S5** Standardised factor loadings, eigenvalues, factor intercorrelations, variance explained and Cronbach’s alpha for 2-factor ESEM model

|  | F1 | F2 |
| --- | --- | --- |
| Physical health | **0.46** | 0.18 |
| Vitality | **0.44** | 0.17 |
| Moral / mood | **0.68** | -0.07 |
| Living environment | **0.52** | 0.02 |
| Memory | **0.34** | 0.19 |
| Relationship with family | **0.40** | -0.13 |
| Relationship with staff | **0.74** | -0.26 |
| Relationship with friends | **0.35** | 0.12 |
| Self-image | **0.53** | 0.18 |
| Keep busy | 0.02 | **0.75** |
| Do things for pleasure | 0.01 | **0.72** |
| Self-care | **0.57** | 0.09 |
| Live with others | **0.43** | 0.14 |
| Make choices | 0.16 | **0.48** |
| Eigenvalues | 4.79 | 1.36 |
| Factor intercorrelations |  |  |
| Factor 1 | - |  |
| Factor 2 | 0.58 | - |
| Variance explained | 22% | 13% |
| Cronbach’s alpha | 0.81 | 0.73 |

**Table S6** Standardised factor loadings, eigenvalues, factor intercorrelations, variance explained and Cronbach’s alpha for 1-factor ESEM model

|  | F1 |
| --- | --- |
| Physical health | 0.59 |
| Vitality | 0.57 |
| Moral / mood | 0.65 |
| Living environment | 0.49 |
| Memory | 0.49 |
| Relationship with family | 0.28 |
| Relationship with staff | 0.51 |
| Relationship with friends | 0.42 |
| Self-image | 0.66 |
| Keep busy | 0.57 |
| Do things for pleasure | 0.54 |
| Self-care | 0.63 |
| Live with others | 0.53 |
| Make choices | 0.59 |
| Eigenvalues | 4.79 |
| Variance explained | 29% |
| Cronbach’s alpha | 0.86 |
